# The New High Resolution Crystal Structure of NS2B-NS3 Protease of Zika Virus

**DOI:** 10.3390/v9010007

**Published:** 2017-01-10

**Authors:** Syed Lal Badshah, Abdul Naeem, Yahia Mabkhot

**Affiliations:** 1 Department of Chemistry, Islamia College University, Peshawar 25120, Khyber Pukhtoonkhwa, Pakistan; 2 National Center of Excellence in Physical Chemistry, University of Peshawar, Peshawar 25120, Khyber Pukhtoonkhwa, Pakistan; naeeem64@yahoo.com; 3 Department of Chemistry, College of Science, King Saud University, Riyadh 11451, Saudi Arabia

**Keywords:** Zika virus, microcephaly, non-structural protein, protease, inhibitors

## Abstract

Zika virus (ZIKV) is the cause of a significant viral disease affecting humans, which has spread throughout many South American countries and has also become a threat to Southeastern Asia. This commentary discusses the article “Crystal structure of unlinked NS2B-NS3 protease from Zika virus” published recently in the journal *Science* by Zhang et al. of Nanyang Technological University, Singapore. They resolved a 1.58 Å resolution structure of the NS2B-NS3 protease of ZIKV and demonstrated how peptide and non-peptide inhibitors interact with this structure, along with the different conformational states that were observed. This protease crystal structure offers new opportunities for the design and development of novel antiviral drugs used for the treatment and control of ZIKV.

## 1. Introduction

Zika virus (ZIKV) is a mosquito-borne virus [1], initially isolated from a monkey in the Zika forest of Uganda [2]. The *Aedes aegypti* (*Ae. aegypti*) mosquito species serves as the principal vector for the virus. ZIKV is a member of the *Flaviviridae* family, which includes other highly pathogenic viruses such as dengue virus, West Nile virus, yellow fever virus, Japanese encephalitis virus, and tick-borne encephalitis virus [3,4,5,6]. ZIKV became endemic in 2015 in South and Central America, with an especially high concentration in Brazil, and from there the virus quickly spread to other parts of the Americas [7,8]. ZIKV crosses the placental barrier in pregnant women, and can cause teratogenic effects, such as microcephaly in newborns, although the exact mechanism is still not fully understood. Due to the dramatic rise in microcephaly cases caused by ZIKV, the World Health Organization (WHO) declared the virus to be a public health emergency [7,9,10,11,12]. The main factors leading to the spread of the virus—and thus increased incidence of microcephaly in newborns—are thought to be the increased mobility of humans and the wide distribution of the *Ae. aegypti* mosquito vector [13,14].

## 2. NS2B-NS3 Protease of Zika Virus (ZIKV)

The genome of ZIKV encodes a single polyprotein that is co- and post-translationally cleaved to generate three structural proteins and seven non-structural proteins [15,16]. Several of the non-structural proteins function as enzymes for the virus [17]. Among these is the protease NS2B-NS3, whose function is to cleave the virus polyprotein at proper sites, and is required for ZIKV replication. Similar to most viruses, the non-structural proteins of ZIKV are suitable drug targets, and it is therefore highly desirable to understand the crystal structure of these non-structural proteins [18].

In their recent article, Zhang et al. resolved a 1.58 Å resolution structure of the NS2B-NS3 protease without a linker [19]. Prior to this, they had also published work on a slightly lower resolution structure with a linker and with different ligands in different states [20,21]. The new unlinked NS2B-NS3 structure has an established binding pocket that does not show prominent conformational changes when a substrate or an inhibitor binds with it. This preformed binding cavity is shaped like a cross and contains sub-compartments, where the different residues of the substrate peptide can bind during catalysis. The NS3 *N*-terminal tetrapeptide group—which contains lysine 14,15, glutamate 16, and glycine 17 (K_14_K_15_E_16_G_17_)—folds into a hairpin structure and occupies this active site or binding cavity. This tetrapeptide forms several different kinds of interaction within the binding pocket, which includes hydrogen bonding and a pi-stacking interaction. Several of the protein intramolecular hydrogen bondings are with the backbone, and that is why it is called the reverse peptide. The formation of the reverse peptide bond is believed to be an ideal area of exploitation for drug design.

In order to understand the full catalytic activity of NS2B-NS3 protease, in vitro activities were performed, and the *C*-terminal part of the ZIKV NS2B was observed to be quite flexible. When the inhibitor is removed from the *C*-terminus of NS2B, it then becomes structurally disordered, and is thusly labeled as an open conformation. On the other hand, the ligand-bound protease is a compact structure, and through folding shows close contact with the NS3, and is labeled a closed form conformation. The previously resolved crystal structure of NS2B-NS3 has a long glycine linker which prohibits ligand binding due to steric clashes of different residues.

The structural dynamics of the NS2B-NS3 protease in solution form were also observed through nuclear magnetic resonance (NMR) spectroscopy, which showed a properly folded form of the protein. The different conformational states of the protease enzyme were explored by titrating it with a bipeptide of acetyl lysine-arginine (AcKR) [19]. The AcKR has been previously shown to act as an inhibitor of the West Nile virus (WNV) protease with an IC_50_ of more than 100 μM [19]. The ^1^H-^15^N-HSQC spectra of ZIKV protease showed different conformational changes upon the dipeptide binding in the catalytic triad and the protease in general [19]. The ^15^N-R_1_, R_2_ and heteronuclear Overhauser effect (NOE) showed stable conformation for the *N*-terminal region and for the *C*-terminal β-hairpin region of NS2B. Zhang et al. also showed that small molecule inhibitors can bind to the ZIKV protease [19] when they resolved a crystal structure of the ZIKV NS2B-NS3 protease with the EN300 molecule ((1H-benzo [*d*] imidazole-1-yl) methanol). Although the EN300 did not directly bind with NS2B, it made several interactions of pi-stacking and hydrogen bonding in one of the pockets of the NS3 part of the protein, and there were no major conformational changes in the protein.

## 3. Concluding Remarks

These results show that the ZIKV protease can be further explored for other active binding pockets, which will be targets for inhibitor molecules. Although Zhang et al. focused on the need to design peptide-based drugs (especially cyclic peptide molecules that act as inhibitors), every option of drug design from small organic molecules to large peptide-based inhibitors should be evaluated for inhibitory interactions with such an influential protease enzyme [19]. Secondly, computational drug design, virtual screening, and molecular dynamic simulation-based approaches are required to determine suitable drug candidates for NS2B-NS3 protease of ZIKV. Similarly, the vaccine development for ZIKV should also be expedited, and the academic and industrial partnership regarding vaccine and therapeutic development needs to be strengthened.
